# Statement of Retraction: Propofol inhibits migration and induces apoptosis of pancreatic cancer PANC-1 cells through miR-34a-mediated E-cadherin and LOC285194 signals

**DOI:** 10.1080/21655979.2024.2360264

**Published:** 2024-05-30

We, the Editors and Publisher of the journal Bioengineered, have retracted the following article:

Hongwei Wang, Hongmei Jiao, Ziru Jiang & Renyi Chen (2020) **Propofol inhibits migration and induces apoptosis of pancreatic cancer PANC-1 cells through miR-34a-mediated E-cadherin and LOC285194 signals**, *Bioengineered*, 11:1, 510-521, DOI: https://doi.org/10.1080/21655979.2020.1754038

Since publication, concerns have been raised about the integrity of data, especially Figure 3, where the figure and the corresponding figure legend do not correspond with each other. Whilst figure 3 appears to be an image of a mouse in a field, the figure legend states:“**Figure 3. Propofol inhibits cell migration by inducing E-cadherin**

PANC-1 cells were transient expression of E-cadherin siRNA or LOC285194 siRNA or its control, then exposure to 20 μg/ml propofol for 72 h. a, E-cadherin protein expression was detected by Western blot assay. b, Cell migration was detected by wound healing assays. vs untreated (24 h), ap<0.01, vs propofol (24 h), bp<0.01, vs propofol (24 h), cp> 0.05.”

Upon investigation, additional concerns about the methodology of the study have also been identified. When approached for an explanation, the authors have not responded to any of our queries about the concerns raised in the article.

As verifying the validity of published work is core to the integrity of the scholarly record, we are therefore retracting the article. The corresponding author listed in this publication has been informed. We have been informed in our decision-making by our Editorial Policies and the COPE guidelines.

The retracted article will remain online to maintain the scholarly record, but it will be digitally watermarked on each page as “Retracted”.

